# Values‐Led Coordination: Connect Coordination Victoria and the Establishment of a State‐Wide Network of Family Carer Mental Health and Wellbeing Connect Centres

**DOI:** 10.1111/hex.70786

**Published:** 2026-08-14

**Authors:** Jessica Phillips, Jennifer Bite, Lisa Casaceli, Clare Fleming, Joanna Tilkeridis, Marie Piu, Melissa Petrakis

**Affiliations:** ^1^ Tandem Melbourne Australia; ^2^ School of Social Work Monash University Melbourne Australia; ^3^ St Vincent's Mental Health Service Melbourne Australia

**Keywords:** caregivers, document analysis, families, lived experience, mental health, reflexive thematic analysis, values‐led

## Abstract

**Background:**

This research sought to identify the values that underscored family carer lived experience coordination of a tailored service for family, carers, and supporters in mental health via analysis of secondary data. We analysed documents related to Connect Coordination Victoria's coordination of the newly established Mental Health and Wellbeing Connect centres (Connect centres) in our state of Australia. Our study asked: *What values can be identified from documents related to Connect Coordination Victoria's family carer‐led coordination of a tailored service for family, carers, and supporters in mental health and what activities demonstrate these values in action?*

**Methods:**

We used document analysis (DA) and reflexive thematic analysis (RTA) to systematically identify, code, and analyse *n* = 41 documents (17,630) words) that captured the coordination activities of Connect Coordination Victoria. To identify the values Connect Coordination Victoria demonstrated, we analysed documents related to two structured Connect Coordination Victoria‐led projects which included Educational Forums, and a Mini‐Showcase event. We sourced documents using a systematic process informed by best practice for document analysis that evaluated each document according to six factors: relevance, authenticity, credibility, accuracy, representativeness, and completeness. A total of 7255 words were included for analysis in this study.

**Results:**

Our document analysis identified (*n* = 4) values guiding Connect Coordination Victoria's coordination of the Connect centres. These values were connection, responsiveness, consistency, and flexibility.

**Discussion:**

Our analysis found that Connect Coordination Victoria's strength as a coordinating body lay first in their status as a family carer‐led coordinating body overseeing the delivery of a tailored service for family, carers, and supporters. And second, in their ability to create spaces that connected an emerging family carer workforce providing a family carer‐led service delivery model. Through identifying the core values of Connect Coordination Victoria by analysing documents related to their coordination, our research emphasised the centrality of values to the practice of the family carer lived experience workforce discipline.

**Conclusion:**

Our research demonstrated a systematic process that coordinators of family carer lived experience workforces and family carer‐led mental health services can adopt to identify and define their practice values for leading and delivering services by family carers, for family carers.

**Patient or Public Contribution:**

This research was developed, designed, conducted, and written by researchers with family carer lived experience. All authors hold a lived experience of caring for and/or supporting people experiencing mental health challenges.

## 1 Introduction

Internationally, vital mental health services are struggling to support consumers and their family, friends, and supporters [1]. In 2019, the Government in our state of Victoria, Australia initiated a Royal Commission into the Mental Health System (RCVMHS) in response to calls from consumers, family, carers, supporters, and health workers that the system was ‘broken’ ([2, 3], p.2). The RCVMHS found that Victorian mental health services are choked by demand that outstrips supply; services are difficult to navigate and stuck in a reactive mode that relies on hospital and bed‐based services; the mental health workforce is under‐resourced and burnt out; services frequently overlook and dismiss lived experience perspectives; and family, carers, and supporters are excluded and denied access to information that could aid them in their caring roles [3]. After hearing over 12,500 contributions, the Commission developed 74 Recommendations now guiding state‐wide system reform. Of the seven principles the Commission developed to steer system reform—underway for five years at the time of writing—two principles concern the needs of family carers: a cohort vital to the mental health system but often ‘marginalised, poorly supported, and misunderstood’ ([4], p.766).

While the RCVMHS took place in Australia, local findings about the need to support the well‐being of family carers reflect well‐established global research. International research has demonstrated, for example, that family carers frequently fill the caring and support gaps left by the ‘inadequacies’ of overwhelmed and under‐funded public mental health systems ([5], p.62; [6, 7, 8]). Family carers often act as the main support for people who experience mental health challenges and while many family carers report benefits from caring, such as companionship and satisfaction, many experience a worsening of their own mental and physical health, social isolation, stigma, blame, loneliness, and financial strain [5, 9, 10, 11]. Further, family carers can feel overlooked by individualistic mental health systems such as those in Australia because within these systems, ‘social life and relationships [can] play a secondary role’ and by consequence, the role of family carers in shaping and aiding recovery can go unacknowledged ([12], p.4; [13]).

In reforming the Victorian mental health system to better meet the needs of family carers, the Commission paved two paths for action. First, the reformed system must provide family carers with better and more tailored supports to thrive in their caring role [14]. And second, the new system must support family carers as *individuals* because both within and beyond their caring roles, they have their own needs, hopes, and wants [14, 15].

To address the need for better and more tailored support for family carers, the Commission recommended the State of Victoria establish eight family carer‐led centres across eight geographical regions, now known as the Mental Health and Wellbeing Connect centres (Connect centres).

### 1.1 The Mental Health and Wellbeing Connect Centres

**Figure 1:**
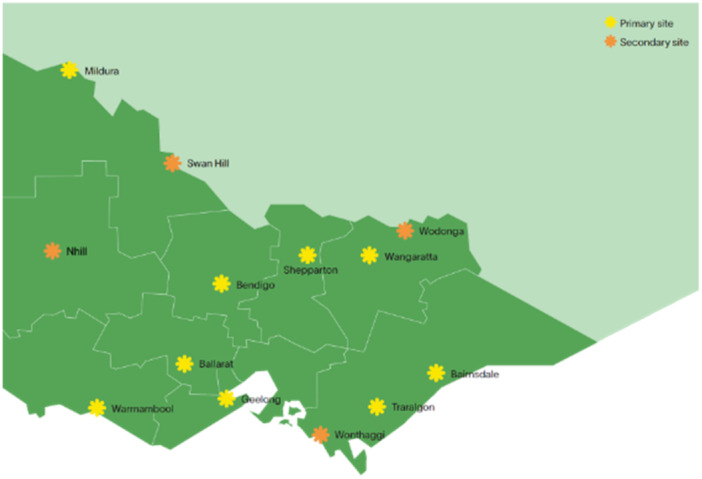
Map of Regional Connect centres created by Tandem, 2025.

**Figure 2:**
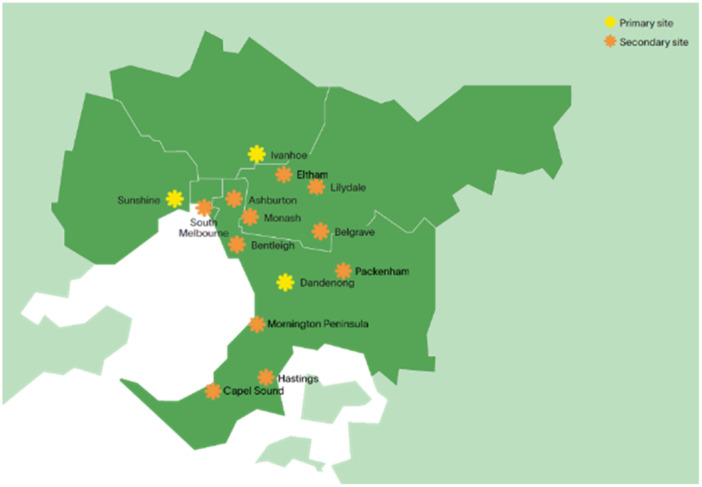
Map of Metro Connect centre locations statewide created by Tandem, 2025.

Across Victoria, eight core Connect centres operate out of eight regions. In addition to these eight main centres, 12 primary sites operate with an additional 15 secondary/satellite sites, making up a total of 35 access points. Together, these sites ensure the needs of family carers are met across a large geographical area (see maps in Figures 1 and 2).

The Connect centres are overseen by lead providers; these providers are local community health services and were appointed by the Victorian Department of Health (DoH) following a competitive tender process. Each lead provider oversees the day‐to‐day operations of their local Connect centre, which includes managing staff recruitment, local service delivery, community outreach, and line managing a majority family carer lived experience workforce (FCLEW).

**Figure 3:**
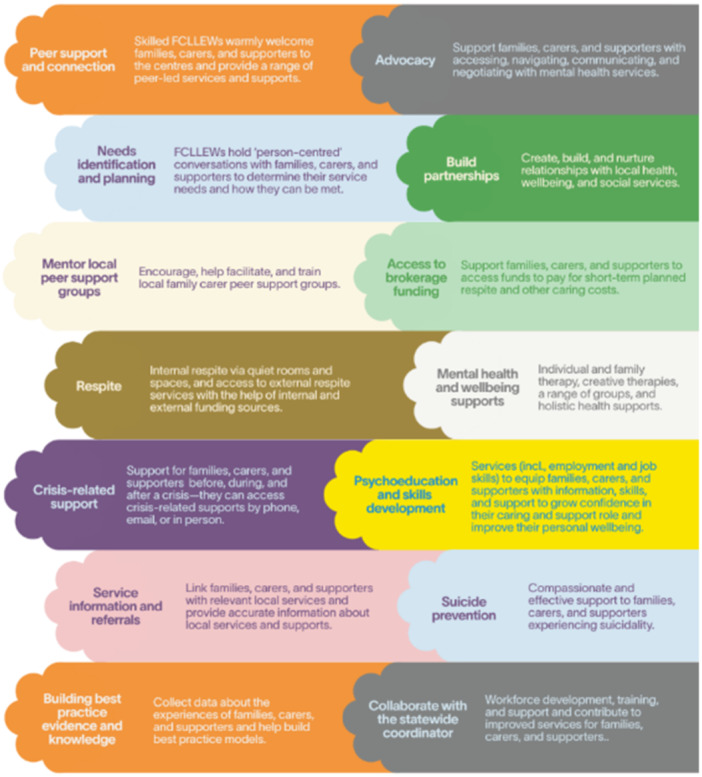
Core service features offered by Connect centres created by Tandem and adapted from [16], Service Development Framework, p. 5–6.

Family, carers, and supporters do not need a referral to access the Connect centres, and they can visit and receive support from a Connect centre in any region. The core service features (illustrated below in Figure 3) capture the guiding values, principles, and objectives of the Connect centres [16].

### 1.2 The Role of Connect Coordination Victoria (CCV) and Tandem

Connect Coordination Victoria (CCV) was established six months after the Connect centres opened in October 2023. The Victorian Government conducted a procurement process in 2023 to identify an organisation to provide statewide support and coordination to the Connect centres: Tandem secured this role.

Tandem was a natural fit for the coordination role because for they have stood as the trusted Victorian peak body for mental health family carers for 30 years (www.tandemcarers.org.au). Tandem also played a pivotal role in establishing the Connect centres by developing the statewide Connect centre service delivery model after the RCVMHS through ongoing advocacy and co‐design activities alongside family carers. Tandem is led and staffed by many people in designated family carer lived experience roles. This family carer lived experience lens ensures the voices of family carers remain central to CCV's coordination of the Connect centres.

### 1.3 Aim

Using document and reflexive thematic analysis methods, our study aimed to identify a set of core values that underpinned the family carer‐led coordination of CCV in providing statewide support to the Connect centres. In this research, we take *values* to mean and include, ‘particular types of beliefs that people hold about what is regarded as worthy or valuable’ and ethical ([17], p.8). Our research aimed to respond to the following question:

What values can be identified from documents related to Connect Coordination Victoria's family carer‐led coordination of a tailored service for family, carers, and supporters in mental health and what activities demonstrate these values in action?

Our research sought to identify concrete examples of CCV's values by analysing documents related to their coordination of the delivery of a tailored service for family carers in mental health. Through our emphasis on family carer values in a coordination context, our research contributes to current projects that seek to understand how best to deliver tailored services for and with family carers, while also supporting the development of an evidence base for family carer lived experience work as a discipline in its own right ([18, 19, 20, 21].

### 1.4 Connect Centre Mental Health Family Carer Lived and Living Experience Workforce (FCLEW)

The Connect centres are staffed by mostly family carers in ‘designated’ lived and living experience roles. For those in designated roles, past and/or present and ongoing experiences of supporting and/or caring for someone experiencing mental health challenges remain a key criterion for employment. As of October 2025, 85.6% of staff across the Connect centres were employed in designated, mostly part‐time roles ([21] p.16). In a recent study which recruited 43% of the Connect centre workforce (*n* = 40 of a possible *n* = 111 staff), 85% of participants (*n* = 34) stated that their Connect centre role was their first designated FCLEW role, 80% (*n* = 32) identified as female, and 80% (*n* = 32) stated they were in an active caring role ([21], p.17–18, 20). While these statistics do not speak for the entire Connect centre workforce, they do suggest that the workforce is an emerging, part‐time, and mostly female cohort who actively care for someone experiencing mental health challenges ([19, 21].

### 1.5 Materials and Methods

#### 1.5.1 Research Design

To identify the core values informing CCV's coordination of the Connect centres, we used two qualitative research methods: document analysis (DA) and reflexive thematic analysis (RTA) [22]. These methods intersected and overlapped in our research.

Reflexive thematic analysis (RTA) made sense as a method because our interest lay in identifying the values CCV led with by systematically analysing written documents detailing their activities. Second, RTA fit well as a method because we wanted this study to feature ‘depth of engagement’ with CCV's activities through a thoughtful, reflexive, and creative approach to meaning making ([22], p.3). Our primary focus was to draw out rich descriptions and interpret the content of the documents to tell a story about CCV's role in coordinating the Connect centres. As this project had a keen emphasis on values, we wanted our methodology to align with our values as family carer researchers, for which RTA very much did, especially in its emphasis on ‘own[ing]’ one's perspective, making deliberate, thoughtful decisions and having a solid presence in our research ([22], p. 1). In the data analysis section to come, we describe how our project aligned with Braun and Clarke's model for RTA.

Bowen (2019) defines document analysis (DA) as a ‘systematic procedure for reviewing or evaluating documents’ (p. 27). DA is a lesser‐known qualitative method that regards secondary documents—meeting agendas and minutes; manuals; background papers; Terms of Reference; books; emails; journals; press releases; application forms; summaries; newspaper clippings; survey data; and programme proposals—as valuable sources of pre‐existing data [23]. DA made sense as a method because we were interested in locating activities that demonstrated values in action based on more than 12 months of CCV coordinating the Connect centres in Victoria. Systematically reviewing documents from the specified date range of October 2023‐August 2025 enabled us to track changes and developments over time from a series of documented activities that we could no longer observe (Bowen 2019). Relying on secondary data reduced the funding required for our research, which took place within a small not‐for‐profit organisation, while granting us more time for ‘data analysis and interpretation’ without compromising on rigour or demanding time from Connect centre staff ([24], p.538).

Our approach to DA and RTA involved three main phases: selection; coding and extracting key quotes; and generating themes. These three core phases aligned with Bowen (2019) and reflect the core phases of RTA detailed by Braun and Clarke [25].

### 1.6 Data Collection

#### 1.6.1 Phase 1: Selecting and Identifying Documents for Analysis

The first phase involved systematically identifying and selecting documents for analysis according to the following six factors (see Table 1) which we adapted from Bowen, 2019 and Morgan, 2022 [26].

**Table 1:** Our criteria for selecting and identifying documents for analysis adapted from Bowen, 2019 and Morgan, 2022 [26].

Factor	Questions for identifying and selecting documents
Relevance	Do the documents relate to CCV and their role in coordinating the Connect centres? Were they produced between October 2023 and 30 August 2025?
Authenticity	Did a member of CCV/Tandem create the document? Can we determine the author/creator? Is the document an original, genuine source of information about CCV?
Credibility	Who created the document, and what is their role in CCV? Does the author hold a conflict of interest? Does the perspective of the article appear to represent some interests at the expense of others? What does the document aim to achieve? How many errors appear in the document?
Representativeness	Does the document reflect the nature and approach CCV took to coordinating the Connect centres (either implicitly or explicitly stated)? Does the document link with the other documents already sourced? Does the document add a new perspective on CCV not covered by other documents or does it offer support to documents already collected?
Meaning	How significant is the content of the document? Does the content read well—understandable, clear, and fit for analysis? Does the document have literal (face‐value, semantic) and/or interpretative (deeper, latent) meaning?
Completeness	Does the document cover CCV in a complete sense, or offer a small window into their coordination role?

The amount of documentation available to analyse was, in plain terms, enormous. To make the analysis achievable within six months to accord with academic mentoring, our primary researcher, JP, under the guidance of M. Petrakis made the decision to draw data from four main projects that represented the core coordination activities of CCV. For this analysis, however, we will only be drawing on data from the Educational Forums and the Mini‐Showcase event because our results were significant and substantial enough to warrant two academic research articles (see Tables 2 and 3). In our companion paper we detail our findings from analysis of the Communities of Practice and the online community co‐esign project.

**Table 2:** Documents selected from the Mini‐Showcase event.

Document type	Word count
Meeting minutes	288
Expressions of interest form for presenters	291
Post‐event survey	1150
Mini‐Showcase programme	243
Mini‐Showcase attendance records	153
Mini‐Showcase run sheet	365
TOTAL words	2490

**Table 3:** Documents selected from the Educational Forums.

Document type	Word count
May 2024: Family violence support options for carers run sheet and information	132
May 2024: Family violence support options for carers survey results	63
July 2024: SHARC working with migrant communities in AOD settings run sheet and information	663
July 2024: SHARC working with migrant communities in AOD settings survey results	62
August 2024: Support for families, carers, and kin who journey alongside someone with BPD survey results	61
September 2024: Eating Disorders Victoria: Connecting carers, responding to and supporting carers of loved ones impacted by eating disorders run sheet and information	525
September 2024: Eating Disorders Victoria: Connecting carers, responding to and supporting carers of loved ones impacted by eating disorders survey results	60
November 2024: Harm Reduction Victoria survey results	62
December 2024: Tuning in to Teens run sheet and information	570
December 2024: Introduction to Tuning in to Teens Tandem notes	552
December 2024: Tuning in to Teens survey results	54
February 2025: Forensicare run sheet and information	604
February 2025: Forensicare survey results	58
April 2025: Victorian Refugee Health Network run sheet and information	244
April 2025: Victorian Refugee Health Network survey results	60
June 2025: Introduction to Carer Perspective Supervision Training run sheet and information	350
June 2025: Introduction to Carer Perspective Supervision Training survey results	58
August 2025: Carer‐inclusive service delivery in mental health run sheet and information	464
Educational forums attendance	123
Total words	4765

In the identification and selection phase, we generated a spreadsheet for tracking the total word count for each document. The spreadsheet contained which criteria each document met and how. Our primary researcher documented her decisions for inclusion/exclusion by creating an audit trail which took the form of a series of transparent, reflective notes that described her decisions [27]. We then asked: how many words and documents do we need for a rigorous analysis? While there was no magic number, the question we pursued in judging how many words and documents to include was, ‘does the data we have provide for and will it substantiate meaningful and significant claims about the coordination values of CCV’? (adapted from [27], p. 841). Given the need to complete the project within six months to accord with academic mentoring support for our research team, we agreed our primary researcher could not analyse more than 10,000 words. A decision was made, in the present study, to analyse findings from the Mini‐Showcase event and the Educational Forums and publish findings from the Communities of Practice and online community co‐design project in a subsequent paper. Below in Tables 2 and 3 we illustrate the documents that met our criteria for inclusion in this present study. In total, we included 7,255 words for analysis and the documents include both qualitative and quantitative data points.

### 1.7 Data Analysis

#### 1.7.1 Phase 2: Coding and Extracting Key Quotes

The second phase involved coding and analysing the selected documents. To code, our primary researcher uploaded all the selected documents to the qualitative cloud‐based software, Quirkos. She then undertook three core actions for each document to progress this phase of the research. These three phases, adapted from Bowen, 2019 and integrating RTA phases included:

1. Skimming the selected documents to identify ‘meaningful and relevant passages of text and data’ (Bowen, 2019, p.32).
2. Rereading the documents, examining their contents in detail, extracting key quotes, and creating a set of initial codes.
3. Refining the codes through subjective interpretation.

Our research adopted what Braun and Clarke refer to as ‘Big Q,’ or qualitative research that takes place within a ‘non‐positivist’ framework ([22], p.1). Positivist frameworks assume that ‘a single tangible reality exists’ and this reality, and knowledge about it, can and must develop ‘objectively without the values [or biases] of the researcher or participants influencing [or interfering with] its development’ ([28], p. 691). In contrast, our research leant into the subjectivity of our primary researcher. We did not employ methods to ensure ‘reliability’ or ‘objectivity’ in the coding phase, such as intercoder agreements, codebooks, or multiple coders ([22], p.2). We had one primary coder, JP, who engaged in reflexive, interpretative practice. She kept a diary while coding, noting her observations and personal reflections and she discussed her reflexive practice regularly with M. Petrakis. We sought a subjective interpretation of the secondary data in the coding phase with the aim (in the subsequent phase) of identifying a set of core values.

#### 1.7.2 Phase 3: Generating Themes

In this phase, we explored shared and united meaning across the data with the aim of telling an ‘interpretative story’ about CCV's approach to coordinating the delivery of a tailored mental health family carer service ([22], p. 2). From her coding in Phase 2, our primary researcher, JP, generated a set of initial themes, which she grouped beneath four values. JP then met with M. Petrakis to discuss her interpretations. Following these discussions, our primary author refined her themes and engaged all co‐authors in two rounds of review to complete the full draft of the manuscript.

### 1.8 Results

#### 1.8.1 Overview

The four themes we developed reflected the core values we identified through our analysis of documents related to CCVs coordination of four primary projects. As noted above, in this paper, we discuss data from two projects (see Tables 2 and 3): the Mini‐Showcase event and the Educational Forums.

### 1.9 Theme 1: Coordinating Spaces to Connect

The primary theme we developed and therefore the defining value of CCV's coordination is captured in the name of both Connect Coordination Victoria and the Mental Health and Wellbeing Connect centres: *Connection*. Our analysis found that CCV's strength as a family carer‐led coordinating body lay in their ability to create spaces that connected an emerging workforce in a brand‐new Victorian service delivery model. The four projects we analysed all aimed at connecting the family carer workforce. However, the Mini‐Showcase (discrete) and the Educational Forums (ongoing) are two interesting and divergent instances of how CCV coordinated spaces for connection and responded to the need the Connect centre workforce had for a range of dedicated spaces to connect.

The Mini‐Showcase was a full‐day, in‐person event held at Melbourne University's Business School in February 2025. Of the four projects we analysed, this event was the only one that invited Connect centre staff to come together and connect in person as a large group, though the event was live streamed online for those unable to attend in person. 60 people attended, including staff from Tandem, the Victorian Government Department of Health (DoH), and the Connect Development Group (CDG). 73% of attendees were Connect centre staff. CCV created and issued a post‐event survey, and 52% of Connect centre staff completed the questions. Members of CCV together with self‐nominated members of the CDG and Connect centre staff formed a working group in September 2024 to co‐plan the logistics for the day.

In the Expressions of Interest (EOI) form inviting Connect centre workers as presenters for the event, CCV described the purpose of the day as ‘showcas[ing] the achievements of the Mental Health and Wellbeing Connect centres […] across Victoria and provid[ing] opportunities for each centre to learn from the innovations and creativity of one another.’ While the explicit purpose of the event was to showcase achievements and learn, the documents we analysed suggested that the implicit purpose and the main impact of the event was for Connect centre staff to connect with one another and with CCV. We saw connection expressed as a guiding value, aim, and overall impact via the presentations and attendees' responses to the event via a post‐event survey.

The content of the Mini‐Showcase presentations demonstrated how central connection was to the work of the Connect centres while the post‐event survey results suggested that dedicated spaces to connect were vital for a workforce who spend their work life designing projects, initiatives, and spaces to connect family carers. It was no surprise our results demonstrated that FCLEWs require the very thing they strive to provide for family carers within a tailored, family carer service.

Seven of the eight presentations at the Mini‐Showcase explicitly detailed an event, initiative, or programme that sought to connect family carers in their local communities. The Grampians Connect centre presented on ‘Co‐design and Community Engagement’ while Loddon Mallee Connect centre described a cruise they organised to ‘celebrate’ and connect family carers during Carers Week 2024. Both Loddon Mallee and Southwest Metro Connect centres spoke to work they have done to build partnerships in their community with complementary health services to better connect family carers with relevant support, while the Northwest Metro and Barwon Southwestern Connect centres presented on in‐house groups they hosted to nurture the creativity and well‐being of family carers.

While the presenters focused on the value of connection for family carers via their ongoing work within the Connect centres, the post‐event survey documents we analysed indicated that the event itself offered Connect centre workers space and time to connect through a dedicated day to ‘meet […] others’ and ‘hear what other centres are doing.’ In the post‐event survey results, Connect centre staff spoke to the value of connecting via ‘learning about the variety of projects and approaches used within the Connect centres.’ Several survey respondents commented on the value of ‘meeting,’ ‘learning,’ ‘hearing,’ ‘seeing,’ and just ‘chatting’ and sharing ‘food’ with colleagues. These terms and the sentiment around connection recurred across the following documented post‐event survey responses: ‘Good to hear what other centres are doing; ‘[i]t was so great to hear what other Centres are doing and how they're using creativity in different ways;’ [i]t was affirming to hear that although we're doing things in slightly different ways, we're still consistent in what we're offering whilst being responsive to the needs of the different geographical regions;’ ‘[m]eeting with the other Connect Centres;’ ‘I loved seeing the different aspects of each centre and the work that has been achieved by all’ and ‘[l]earning what other Connect centres do and what they are planning.’

### 1.10 Theme 2: Responding Enhances Connection

The positive post‐event survey results for this event, coupled with the strong attendance records demonstrated that the CCV‐coordinated Mini‐Showcase event was both engaging and enjoyed by Connect centre staff because it offered them something that can still feel rare, yet vital in a post‐covid context: opportunities to gather in person with colleagues and connect over shared interests and a collective passion for ‘supporting carers.’ The themes of Coordinating Spaces to Connect and Responding Enhances Connection linked here because while coordinating opportunities for connection, our analysis found that CCV also *responded* to a vital need the Connect centre workforce had *to* connect, especially in person.

In the post‐event survey documents, one respondent summed up the rarity of these in‐person opportunities and their value. When writing about the significance of the day and what was enjoyed most, they noted in their survey response that ‘being able to connect with each other in person’ was the most satisfying part of the event. ‘I know these opportunities are limited’ they wrote, ‘but the face‐to‐face contact really grounds the work we're doing and the relationships we're building with each other to feel that we are a united initiative.’

The vital importance of dedicated, in‐person spaces for the Connect centre workforce to connect was perhaps best emphasised by one attendee in the post‐event survey responses we analysed who, when responding to a question about the suitability of the venue for the event, framed their response in terms of how to best organise the room in future to maximise opportunities to connect. ‘I liked the venue’ they wrote, ‘however, I am not sure about the theaterette style […] I think a more open room, such as one set up in a cabaret style may have been more conducive to additional networking […].’ 91% of survey respondents recommended holding a similar event next year, which suggested that CCV coordinated an important space for connection, and thereby responded to a need family carer Connect centre workers have for in‐person spaces to connect.

In contrast to the discrete, in‐person Mini‐Showcase event, the Educational Forums were held online each month and featured a guest presenter(s) from a relevant Victorian mental health service. The main purpose of the Educational Forums, as evidenced by the recurring, stable question on the post‐session surveys, was to boost the confidence of the Connect centre workforce to respond to the diverse needs of family carers seeking support from the Connect centres. In this way, while delivered to the Connect centre workforce, the CCV‐coordinated Educational Forums aimed to equip the workforce to deliver an informed, tailored service to family carers. For example, the session topics supported Connect centre staff to navigate specific mental health family carer circumstances such as the ‘criminal justice space,’ ‘eating disorders,’ ‘refugee and asylum seeker communities,’ and ‘family violence.’ While these CCV‐coordinated sessions were delivered online in a webinar‐style format with fewer opportunities beyond dedicated question time to informally connect, they fostered and expressed connection as a vital value in two crucial ways. First, the sessions were consistent in their form and were held regularly. Our analysis of secondary documents related to the Educational Forums demonstrated that they began in April 2024 and continued at the time of writing. The sessions occurred most months and attendance remained steady across the 10 sessions we analysed (see Table 4). These Educational Forums adhered to a similar format (introductions, recognition of family carer lived and living experience, housekeeping, presentation, and question time) and provided a stable, regular point of connection between Connect centre staff, CCV, and the Victorian mental health system. The consistency of the Educational Forums suggests that regular, ongoing spaces for Connect centre workers can and do foster connection while also building the capacity of the workforce to deliver a consistent service to family carers.

**Table 4:** Educational F4orum post‐session survey results, June 2024–August 2025.

Session date	Topic/presenters	Attendance	Do you feel more confident?	Would you recommend this session to a colleague?	Was this session relevant to your role?
May 2024	Family violence support options for carers	N/A	Very confident: 1 Confident: 8 Somewhat; 1 No change: 1 Less confident: 0 No change: 0	Yes: 11 No: 0	Yes: 11 No: 0 Not sure: 0
July 2024	SHARC: Working with migrant communities in AOD settings	44	Very confident: 0 Confident: 4 Somewhat: 2 No change: 0 Less confident: 0 Confused: 0	Yes: 6 No: 0	Yes: 6 No: 0 Not sure: 0
August 2024	Support for families, carers, and kin who journey alongside someone with BPD	45	Very confident: 0 Confident: 2 Somewhat: 2 No change: 0 Less confident: 0 Confused: 0	Yes: 5 No: 0	Yes: 5 No: 0 Not sure: 0
September 2024	Eating Disorders Victoria: Connecting Carers: Responding to and supporting carers of loved ones impacted by eating disorders	31	Very confident: 0 Confident: 1 Somewhat: 2 No change: 0 Less confident: 0 Confused: 0	Yes: 3 No: 0	Yes: 3 No: 0 Not sure: 0
November 2024	Harm Reduction Victoria	22	Very confident: 1 Confident: 1 Somewhat: 2 No change: 1 Less confident: 0 Confused: 0	Yes: 5 No: 0	Yes: 4 No: 0 Not sure: 1
December 2024	Tuning in to Teens	30	Very confident: 0 Confident: 3 Somewhat: 1 No change: 0 Less confident: 0 Confused: 0	Yes: 3 No: 0	Yes: 3 No: 0 Not sure: 0
Feb 2025	Forensicare	51	Very confident: 1 Confident: 6 Somewhat: 7 No change: 2 Less confident: 0 Confused: 0	Yes: 16 No: 0	Yes: 15 No: 0 Not sure: 0
April 2025	Victorian Refugee Health Network (VRHN)	54	Very confident: 2 Confident: 5 No change: 1 Less confident: 0 Confused: 0	Yes: 10 No: 0	Yes: 10 No: 0 Not sure if appropriate: 0
May 2025	Noticing the Signs, Navigating the Risks: Family Violence education for Connect centre workers (repeat screening from May 2024 forum)	17	N/A^3^	N/A	N/A
June 2025	Introduction to Carer Perspective Supervision Training	29	Very confident: 1 Confident: 1 Somewhat: 0 No change: 0 Less confident: 0 Confused: 0	Yes: 2 No: 0	Yes: 2 No: 0 Not sure: 0
August 2025	Carer‐inclusive service delivery in mental health^4^	42	Good understanding: 7 Some understanding: 3 No change: 1 Confused: 0	Yes: 11 No: 0	Yes: 11 No: 0 Not sure: 0

Second, these Educational Forums fostered and expressed connection as a value via CCV's organisation of a diverse range of presenters as evidenced via the run sheets and information documents compiled by CCV for each session. These sessions performed an important connecting function for a newly established workforce in a brand‐new service model by linking them with established services and the people behind these services. For example, the run and information sheets demonstrated that Connect centre staff were able to meet and learn from family peer support workers with sevent years’ combined experience working in child and youth mental health services as well as lived experience experts employed in forensic mental health services, and a lived experience carer consultant with over 17 years’ experience working to improve ‘the family carer experience in MH services.’ The chosen presenters, as documented in these information sheets, were shown to hold extensive family carer lived experience and lived expertise, making them valuable, relatable mentors for a newly established Connect centre workforce seeking knowledge and skills in how to respond to a diverse range of family carers.

The themes of Coordinating Spaces to Connect and Responding Enhances Connection blended in the case of the Educational Forums because in coordinating online spaces for Connect centre workers to come together monthly to learn and connect with experienced mental health workers, CCV responded to the needs of the family carer workforce to participate in these dedicated learning spaces. The post‐event survey responses (see Table 4) indicated that the Educational Forum sessions both increased the confidence of those that attended while feeling relevant to their day‐to‐day work with family carers in a tailored service. Likewise, as the survey results below indicate in Table 4, Connect centre staff unanimously recommended their peers watch recordings of the sessions if they were unable to attend. These ‘would you recommend’ figures are significant because a strong willingness to recommend the sessions to others demonstrates that those that attended found them useful and believed in their utility and relevance enough to take some accountability if their peers did not find them useful.

### 1.11 Theme 3: Consistent Coordination Builds Structures for Trust

Across the four projects we analysed, CCV enacted repeated rituals and processes. These rituals and processes, often led by the same people within CCV, structured individual engagements and established patterns across CCVs engagement with Connect centre staff.

The most consistent ritual we witnessed CCV enacting across the secondary data we analysed for the two projects discussed here was a Recognition of Family Carer Lived and Living Experience (Recognition of FCLLE). This statement was issued at the beginning of each Educational Forum, and the Mini‐Showcase event. Table 5 displays the consistency of this ritual across CCV‐coordinated projects.

**Table 5:** Occurrence of recognition of FCLLE.

Project	Number of documents analysed for this project	Number of times a Recognition of FCLLE took place across the documents analysed
Mini‐Showcase event (incl., planning)	7	1^5^
Educational Forums	19	8

To illustrate the form and content of a Recognition of FCLLE, take this example delivered by the CCV Chair of an Educational Forum session on 24 July 2024:

I want to recognise those with lived and living experience of walking alongside someone with mental health challenges and those with their own personal journey of mental health challenges. This experience and knowledge brings invaluable understanding and compassion to our lives and to our everyday encounters with supporters, families, kin and friends in our work lives.

This ritual is significant for the FCLEW across the Connect centres because it explicitly and repeatedly acknowledges, recognises, and therefore elevates the value of their lived and living experiences in their roles supporting family carers within a tailored service delivery model. This intentional recognition foregrounds family carer lived and living experience by making visible both the workforce's ‘invaluable knowledge and experience’ and the primary aim of their role: to lean on that experience to deliver a high‐quality service to family carers. While more research is needed to understand the direct impact of this ritual on the experiences of FCLEWs within the Connect centres, we note that this ritual could plant the seeds for trust while fostering compassionate working relationships between CCV and the Connect centre workforce because this act values, includes, and acknowledges the often invisible but nonetheless formative experiences that drive FCLEWs to do they work they do. These acts of recognition also express gratitude by directly connecting lived and living experiences of caring with improving the lives of family carers via compassion and understanding. Our forthcoming paper discusses consistency in more depth across the CCV‐led Communities of Practice and online community co‐design project.

### 1.12 Theme 4: Flexibility Sews Elastic Spaces

We found that while many of the spaces CCV coordinated for Connect centre staff were structured to varying degrees, they were also elastic spaces, capable of stretching and accommodating their changing needs.

CCV expressed flexibility in the case of the Mini‐Showcase in so far as the event was held in person, but ‘live streamed and recorded’ to accommodate all Connect centre workers regardless of their ability to attend on the day. We also saw flexibility at work, for example, in the case of the Educational Forums where each one hour session could extend beyond that hour ‘if discussion is rich and the speaker is available, acknowledge[ing] that some people may need to leave, but that you will stay online for any final questions.’ These sessions were also recorded and posted online for Connect centre workers to rewatch at a convenient time.

We witnessed the desire to stretch and accommodate to respond to the needs of Connect centre staff via the frequency and number of surveys delivered to staff to learn more about their needs, preferences, and experiences. Table 6 illustrates the kinds of surveys conducted for each project we analysed between October 2023 and August 2025. Responses to these surveys were limited, however, and while the results of our secondary data analysis were promising and illuminating, more research is needed to determine the degree to which CCV was responsive to the concerns and issues raised via these surveys. We discuss flexibility in greater depth in our forthcoming paper.

**Table 6:** Surveys conducted by CCV across Mini‐Showcase event and Educational Forums.

Project	Survey	Number of respondents
Mini‐Showcase	Post‐event survey Feb	*n* = 23
EducationalForums	Post‐forum survey May 2024	*n* = 11
	Post‐forum survey July 2024	*n* = 6
	Post‐forum survey August 2024	*n* = 5
	Post‐forum survey September 2024	*n* = 3
	Post‐forum survey November 2024	*n* = 5
	Post‐forum survey December 2024	*n* = 4
	Post‐forum survey February 2025	*n* = 16
	Post‐forum survey April 2025	*n* = 10
	Post‐forum survey June 2025	*n* = 2

### 1.13 Discussion

In this research, we developed a systematic process to identify the core values of CCV through analysing secondary data related to their term coordinating the newly established Mental Health and Wellbeing Connect centres for family carers in Victoria, Australia. Our analysis found that CCV's strength as a coordinating body lay first in their status as a family carer‐led coordinating body overseeing the delivery of a tailored service delivery model for family carers. And second, in their ability to create structures and spaces that connect the Connect centre workforce—an emerging family carer workforce in a brand‐new Victorian service model. Our results suggest that CCV embraced connection as a guiding value through creating dedicated, peer‐led, in‐person and online spaces that united and strengthened the family carer‐led identity of the Connect centres and helped to foster, as one survey respondent said, when rcompleting the Mini‐Showcase post‐event survey, a much needed ‘feeling of collective community.’

### 1.14 Implications for Policy

Our research identified consistency as a core coordinating value of CCV. In our research, we paid special attention to the consistency of recognition's of FCLLE across CCV's activities. We demonstrated that these recognitions elevated family carer lived experience and therefore stood in direct contrast to the lived reality of many family carers who have routinely felt like ‘invisible experts’ when ‘devalued,’ ‘excluded’ and ‘dismissed’ by mental health systems that can, at worst, blame them for the mental ill health of the consumers they care for ([8], p.615).

Our finding regarding consistency suggests that consistent leadership can create a stable environment for family carers, FCLEWs, and for the providers overseeing the daily operations of individual centres in their local regions in our state of Australia. A stable environment matters here because it could help to ensure that many of the family carer recommendations from the RCVMHS, together with current CCV‐led FCLEW initiatives and emerging, evidence‐based recommendations can be implemented effectively and efficiently across the Connect centres ([19, 21]

Consistent coordination across the Connect centres promotes the delivery of a stable service for family carers—a vulnerable cohort who, historically, have not received consistent support from mental health providers. Research indicates that family carers nominate ‘ongoing support’ as one of their most important needs from mental health service providers ([10], p.8). Consistent, stable, family carer‐led coordination of a tailored service for family carers is, in other words, vital for the well‐being of family carers and the longevity of the Connect centre service model.

Given the newness of the Connect centre service model in our state of Victoria, consistent, ongoing funding and family carer lived experience coordination from a trusted body like CCV could support the longevity of the service delivery model. This recommendation aligns with two recent Australian Government inquiries.

The Productivity Commission's Inquiry Report into the Mental Health System [29] acknowledged that within the current Australian mental health system, funding for services like the Connect centres and CCV ‘is fragmented and based on short contract cycles which make it harder to deliver quality services on a continuous basis to people’ (p. 55). The RCVMHS Final Report (2021) echoed this claim when they stated that ‘funding certainty of at least 5 years is necessary in the reformed system to ensure consistent and sustained levels of advocacy’ and mental health service provision across the state (Volume 3, p. 78). Consistent, ongoing care, support, and service delivery matters for mental health family carers locally and internationally because many report living in a perpetual state of uncertainty, instability, and ‘frustrated waiting’ while the person they care for deteriorates enough to become eligible for hospital‐based crisis care ([8, 30], p.139). Our study highlights the importance of consistency in the family carer‐led coordination of the Connect centres because stable coordination ultimately supports the delivery of an ongoing, reliable service for mental health family carers.

### 1.15 Implications for Practice

Much of the international research to date has focused on family carer lived experience workers in frontline roles such as family carer peer work (e.g.,: [31, 32, 33, 34]). Yet, family carer lived experience workers are employed across policy, research, leadership, and as we have shown, in coordination roles like CCV. Our research builds upon this important, growing body of discipline‐specific research by identifying the values enacted by CCV in coordinating the delivery of a tailored service for family carers in mental health. Regardless of the form FCLEW takes, the discipline remains informed by solid values and principles that guide practice.

To grasp the ‘why’ behind the values of FCLEWs we only need return to the challenges faced by family carers we described in our introduction. As discussed, family carers can feel excluded and dismissed by mental health systems that view them, in some cases, as ‘the enemy’ ([24], p. 83; Wyder et al., 2021; [8]). Within individualistic mental health systems, the unique well‐being needs, insights, concerns, and vital support role played by family carers can get overlooked [8, 13, 24]. These common, shared, collective challenges among family carers give rise, in part, to the foundational values of the FCLEW discipline. For example, the recently published Mental Health Family Carer Workforce Discipline Framework sets out that FCLEW workers promote values such as relationality, inclusivity, and equity through their practice ([20], p. 23‐24).

Through identifying the core values of CCV through analysing documents related to their coordination, our research emphasises the centrality of values to the practice of the family carer lived experience workforce discipline. A values‐led approach to practice ensures that services like the Connect centres can maintain a strong identity that prioritises the health and well‐being of family carers. Those looking to implement a similar family carer‐led service model including a coordination body like CCV, could consider identifying the values that underscore their practice through a similar approach to the one modelled here. We have demonstrated a systematic process by which others can identify their values, while also suggesting that values such as connection, responsiveness, consistency, and flexibility—together with the foundational values of the FCLEW discipline as described in the Discipline Framework (2025)—are significant and significantly important for coordinators to hold when supporting the delivery of a mental health service by family carers for family carers.

### 1.16 Limitations

The detailed record keeping of CCV made this research possible. While we extracted 7255 words for analysis in this present study, these data are, however, a secondary source of information about CCV. Additional primary data could have included interviews with Connect centre workers. In future, we look forward to building upon these preliminary findings via subsequent fieldwork and interviews.

### 1.17 Conclusion

In systematically identifying the values of CCV, our research detailed a process others can adopt to identify their practice values from secondary data and exsiting documents related to their core activities. Our research demonstrated that coordinators of services for family carers in mental health, especially in newly established service delivery models like the Connect centres, could benefit from systematically and intentionally identifying their practice values to ensure their values continue to inform the quality of care family carers receive.

## Author Contributions

**Jessica Phillips:** conceptualisation, methodology, writing – review and editing, writing – original draft, investigation, formal analysis, project administration. **Jennifer Bite:** conceptualisation, writing – original draft, writing – review and editing. **Lisa Casaceli:** writing – original draft, writing – review and editing, validation. **Clare Fleming:** writing – original draft, writing – review and editing, validation. **Joanna Tilkeridis:** project administration, writing – review and editing, writing – original draft, supervision. **Marie Piu:** conceptualisation, writing – original draft, writing – review and editing, supervision. **Melissa Petrakis:** conceptualisation, investigation, writing – original draft, writing – review and editing, formal analysis, methodology, validation, supervision.

## Conflicts of Interest

Jennifer Bite left her role at Tandem part‐way through the research project to commence work at the Victorian Department of Health. The views expressed by Jennifer Bite here reflect her work with CCV and Tandem, and not the Department of Health. There are no further declared conflicts of interest.

## Data Availability

The data that support the findings of this study are available from the corresponding author upon reasonable request.
